# Development of a universal measure of quadrupedal forelimb-hindlimb coordination using digital motion capture and computerised analysis

**DOI:** 10.1186/1471-2202-8-77

**Published:** 2007-09-18

**Authors:** Lindsay Hamilton, Robin JM Franklin, Nick D Jeffery

**Affiliations:** 1 Brain Repair Centre and Department of Veterinary Medicine, University of Cambridge, Madingley Road, Cambridge, CB3 0ES, UK

## Abstract

**Background:**

Clinical spinal cord injury in domestic dogs provides a model population in which to test the efficacy of putative therapeutic interventions for human spinal cord injury. To achieve this potential a robust method of functional analysis is required so that statistical comparison of numerical data derived from treated and control animals can be achieved.

**Results:**

In this study we describe the use of digital motion capture equipment combined with mathematical analysis to derive a simple quantitative parameter – 'the mean diagonal coupling interval' – to describe coordination between forelimb and hindlimb movement. In normal dogs this parameter is independent of size, conformation, speed of walking or gait pattern. We show here that mean diagonal coupling interval is highly sensitive to alterations in forelimb-hindlimb coordination in dogs that have suffered spinal cord injury, and can be accurately quantified, but is unaffected by orthopaedic perturbations of gait.

**Conclusion:**

Mean diagonal coupling interval is an easily derived, highly robust measurement that provides an ideal method to compare the functional effect of therapeutic interventions after spinal cord injury in quadrupeds.

## Background

During the past 10–15 years, neuroscientists have provided statistical evidence for efficacy of many novel interventions in ameliorating the loss of function that accompanies spinal cord injury (SCI) in proof-of-concept experiments in rodent models (see reviews [1,2]). These data prompt asking whether any of these interventions can be successfully translated into therapeutically valuable treatments for clinical SCI patients [3,4]. One response has been to initiate immediate investigations in human patients and several groups throughout the world have already commenced clinical trials on various cell transplant strategies [5-7]. Indeed, progress towards application in human patients has been sufficiently rapid that a series of guidance articles has recently been published to ensure results of clinical trials are readily interpretable [8-10].

However, there remains a recognised need for further investigations in non-human species, especially with regard to cell transplantation repair therapies [11], such as bone marrow stromal cells [12-14], Schwann cells [15,16] and olfactory ensheathing cells [17,18]. Such extended testing might also be of benefit for screening many potential pharmacological interventions such as chondroitinase ABC [19], or combinations of therapies [20,21] prior to application in humans. These animal studies can address the potential obstacles in translating success in treatment of small, uniform lesions in experimental rodents into similar results in large, heterogenous lesions in human clinical patients. The inevitable heterogeneity of clinical cases provides the most difficult potential obstacle to detection of clinical efficacy, and thus implementation in humans patients, yet constitutes the most probable reason for failure of therapies to translate from lab to clinic [22].

Spinal cord injury (SCI) is common in domestic dogs and provides a useful large cohort of clinical patients, with a comparable degree of lesion heterogeneity to their human counterparts, in which therapies can be screened prior to introduction into human patients. Preliminary studies have confirmed the usefulness of this approach and the safety of olfactory ensheathing cell (OEC) transplantation in the dog [23]; robust, quantitative methods of functional outcome analysis for clinical SCI are now required for progression to Phase II trials in this species.

There are many methods for analysing the functional outcome after SCI in experimental rodents, including both categorical and numerical scores based on various types of observation (reviewed by [24]), most notably the widely-used BBB score [25]. Of specific relevance are reported numerical methods by which coordination between fore and hind limbs can be accurately quantified and correlated with the severity of experimental rodent SCI [26,27]. Whilst potentially applicable to dogs, these methods are time-consuming and potentially liable to failure when applied to analysis of locomotion of dogs of variable size and conformation.

The widespread availability of highly sophisticated motion capture and analysis equipment, such as high speed video images acquired from infrared cameras, has revolutionised the ability to very precisely analyse kinetic and kinematic disturbances in the dog (reviewed by [28]). Moreover, domestic dogs are excellent candidates for motion analysis since they are highly amenable to training and are relatively large, which facilitates limb labelling. However, a drawback is their great variability in size and conformation meaning that gait patterns (*e.g*. between the walk, trot, pace *etc*) may vary between different uninjured patients, thus hindering easy comparison of outcome between groups of heterogenous individuals. Additionally, domestic dogs suffer a wide range of orthopaedic conditions, any of which may impact on the kinematics of their gait; and, since the presence or extent of such conditions are highly unlikely to be known at the time of injury, may subsequently confound attribution of gait abnormalities to SCI.

Our aim in this study was to use digital video motion capture equipment and modern computerised analysis to devise a simple numerical parameter to describe forelimb-hindlimb coordination that would be independent of size, conformation, speed of movement or pattern of gait and therefore could be applied to all quadrupeds.

## Results

### Normal dogs

#### Step cycle duration (see Fig. 1)

**Figure 1:**
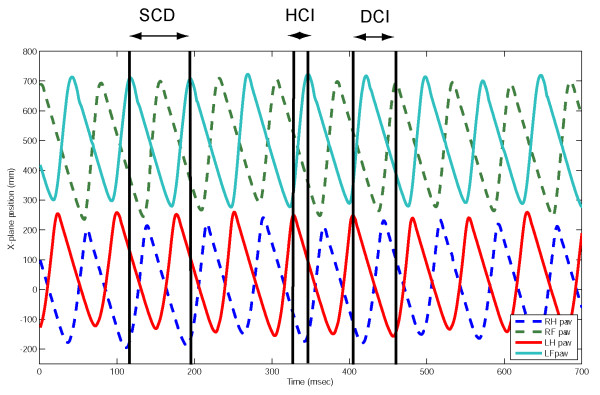
QTM plot illustrating sine wave-like movement of fore and hind paws in the x-plane during treadmill walking in a normal dog. SCD indicates the step cycle duration, measured between consecutive treadmill contact by the left forelimb paw; HCI indicates the left homolateral coupling interval, measured between consecutive contact between the left hindlimb and left forelimb paws; DCI indicates diagonal coupling interval, measured between treadmill contact made by the left hindlimb paw and the right forelimb paw.

We first measured step cycle duration to provide a baseline against which other gait parameters could be normalised for comparison between individuals of different sizes and conformation and between groups of dogs with differing lesions. A mean of 64 step cycles (range 30–120) were accepted for analysis (see Methods) from dogs in this group (the number of steps examined from animals in each group were statistically equal: ANOVA: F(2,30) = 1.46; p = 0.25). We defined *step cycle duration *as the time interval between initial contact with the treadmill belt by one forelimb paw and the subsequent contact by the same paw. The step cycle duration was variable, with small fluctuations occurring between individual steps within each recording session at a specific speed. Between dogs, step cycle duration was shorter at any given speed for smaller individuals, but similarly-sized dogs did not necessarily have similar step cycle durations at the same treadmill speeds. Despite this, even without correction for the variation in speed of walking between individuals, there was, as would be expected, a significant correlation between step cycle duration and length of the tibia in these normal dogs (Pearson's correlation test; r = 0.741; p = 0.0091). Statistical analysis revealed that tibial lengths were indistinguishable amongst all analysed groups of dogs (ANOVA; F(2,30) = 1.07; p = 0.36).

#### Limb coupling (see Fig. 1)

Since this study was focussed on discovery of a simple parameter that would quantify the coordination between forelimbs and hindlimbs, we next investigated the timing between placement of specific pairs of paws on the treadmill. We defined *homolateral coupling *as the time between contact of the paws of the forelimb and the hindlimb on the ipsilateral side and *diagonal coupling *as the time between contact on the treadmill belt of the forelimb paw on one side and that of the contralateral hindlimb paw. Again, we found both parameters to vary considerably between individual dogs, although the coupling intervals remained a consistent proportion of each dog's step cycle duration at a specified speed, confirming that the step cycle duration was the same for each of the limbs. The duration of limb coupling intervals as a proportion of the overall step cycle changed with speed for each dog, with some individuals changing their homolateral coupling intervals, indicating a change of gait pattern towards 'pacing' (*i.e*. simultaneous, or near simultaneous, placement of the homolateral fore and hindpaws). However, at any specified speed the coupling intervals remained constant for each individual dog.

**Figure 2:**
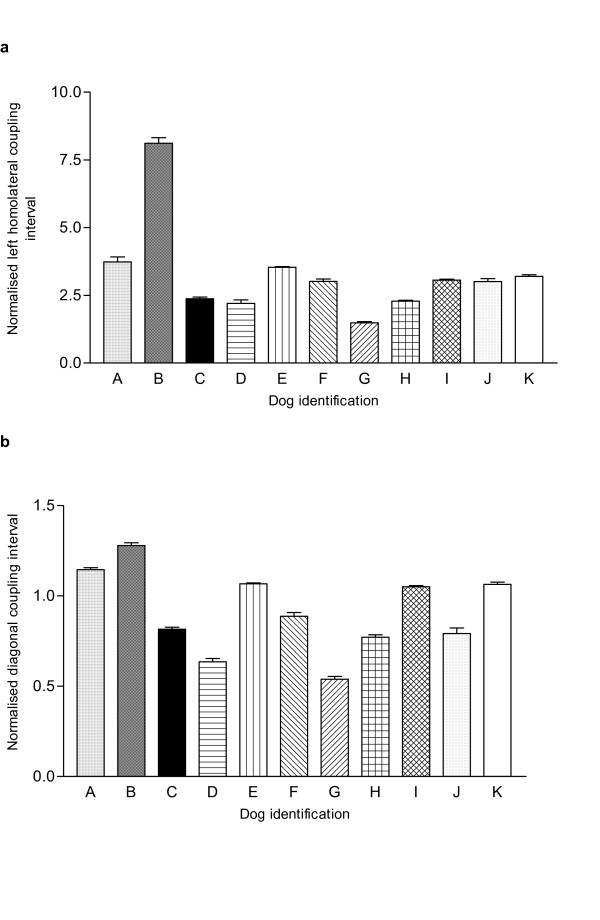
Histograms illustrating the variability in normalised (**a**) left homolateral and (**b**) diagonal (left hind-right fore) coupling intervals between normal individuals. One-way ANOVA confirms a significant difference amongst the individuals [for lateral coupling: F(10,681) = 267.7; p < 0.0001; for diagonal coupling: F(10,684) = 198.9; p < 0.0001). Tukey's *post hoc *tests confirmed significant difference in this parameter between many specific pairs of dogs.

In an attempt to permit comparison between individuals we normalised the limb coupling times by expressing them as a proportion of the step cycle duration. However, comparisons between individuals revealed persistent significant differences [ANOVA tests, left homolateral coupling: F(10,681) = 267.7; p < 0.0001; left hind/right fore diagonal coupling: F(10,684) = 198.9; p < 0.0001]. Further visual comparison of the limb coupling plots (Fig. 2), suggested that this resulted from adoption of gait patterns that varied between individual dogs, illustrating an additional layer of complexity to be overcome in future comparisons made between SCI and normal dogs.

#### Mean diagonal coupling interval (see Fig. 3)

**Figure 3:**
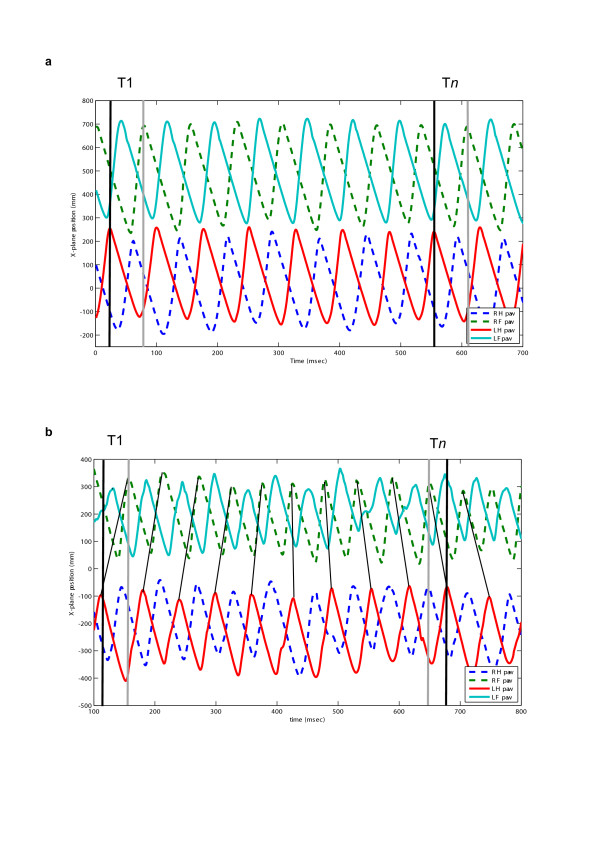
QTM plot to illustrate the calculation of the mean diagonal coupling interval. T1 illustrates the interval between left hindlimb and right forelimb paw touching the treadmill at the beginning of the recorded sequence and T*n *illustrates the interval between the same limb pair at the end of this (short) sequence. **a**: in a normal dog there is ~0 time difference between T1 and T*n*. **b**: in a SCI dog there is a large change between T1 and T*n *– note that the hindlimb paw movement is considerably delayed. The lines between forelimb and hindlimb plots indicate sequential diagonal pairs and highlight the change in temporal sequence during the recording period, which in this instance is sufficient to alter the sequence of hind and fore paw contact within the period of this recording. The mean diagonal coupling interval is derived by dividing the total difference between T1 and T*n *in a sequence by the number of steps (see Methods).

In order to provide a more robust measure of the rigidity of coordination between the forelimbs and hindlimbs we devised a summary descriptive measure of coordination using matrix analysis software. *Mean diagonal coupling *interval was defined (as described in the Methods) as the average change in diagonal coupling interval per step during an entire recording session for each dog.

When thus calculated all our normal subjects had a mean diagonal coupling interval of ~0; the values were expressed as deviation from zero (for ease of subsequent statistical testing) and ranged from 0 to 0.267.

### Spinal cord injured dogs

We next investigated how these parameters were affected by thoracolumbar spinal cord injury (SCI). All the dogs with spinal cord injury in this study had lesions between T11 and L2 spinal cord segments (Table 1). Although the causes of spinal cord injury were different between individuals, all were unable to walk without support during this study period. Therefore, in order to assess the coordination between forelimbs and hindlimbs the hindquarters were maintained in position on the treadmill by using a support band under the abdomen (see Methods). All SCI dogs had movement of the hindlimbs in the x-plane that was independent of passive movement elicited by the treadmill and a mean of 73 step cycles (range 30–170) was accepted for analysis in dogs in this group. However, the kinematics of hindlimb intralimb motion varied between dogs, most noticeably in vertical (y-plane) movement; some dogs dragged the paws on the treadmill during the 'swing' phase, whilst others lifted the paws inappropriately high.

**Table 1:** Clinical details of spinal cord injured dogs

Dog ID	Tibial length	Lesion type & location	Lesion severity
1	11	T11/12 fracture	Incomplete
2	20	T13/L1 nephroblastoma	Incomplete
3	19	T12/13 acute IVD	Incomplete
4	15	L1/L2 acute IVD	Incomplete
5	7	L1/L2 acute IVD	Incomplete
6	7.5	L1/L2 acute IVD	Complete
7	8	T13/L1 acute IVD	Incomplete
8	22	L1/L2 acute IVD	Complete
9	15	T13/L1 acute IVD	Complete
10	9.5	L1/L2 acute IVD	Incomplete
11	11	T12/13 acute IVD	Complete
12	14	T12/13 fracture	Complete

Note: dogs have 13 thoracic and seven lumbar vertebrae.

**Figure 4:**
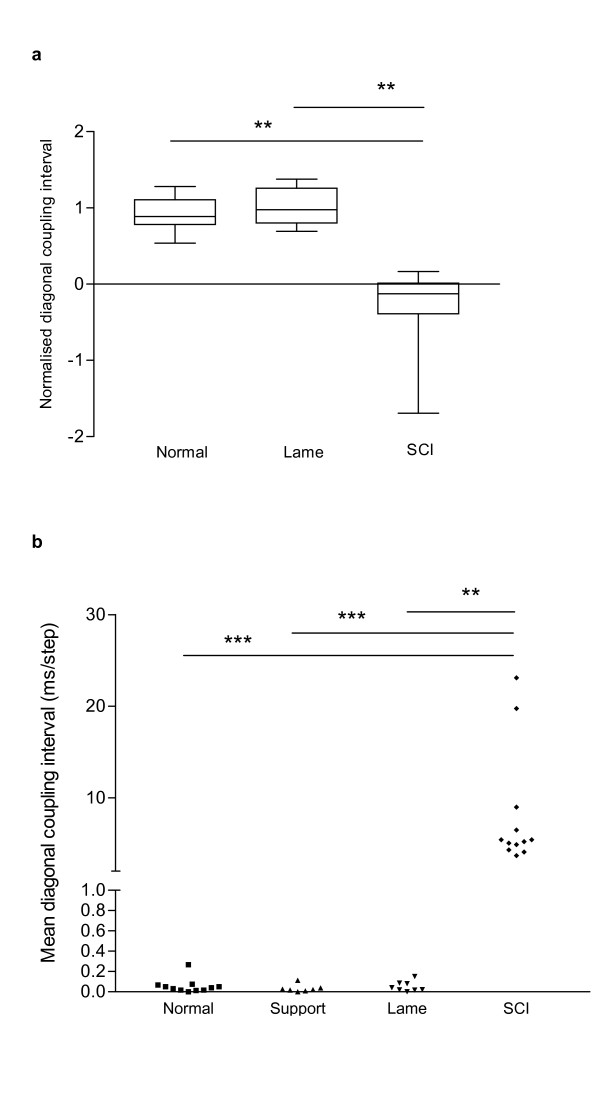
**a**: Box and whisker plot illustrating normalised diagonal coupling intervals in normal, lame and SCI dogs. Kruskal-Wallis test revealed a significant difference (p < 0.0001) amongst the groups; post hoc testing confirmed a significant difference between SCI dogs and both other groups (Dunn's multiple comparison tests, p < 0.001) but no difference between lame and normal dogs. **b**: Scatterplot of the mean diagonal coupling intervals in normal dogs with and without abdominal band support, lame and SCI dogs. Kruskal-Wallis test revealed a significant difference (p < 0.0001) amongst the groups; there were significant differences between SCI dogs and all other groups (Dunn's multiple comparison tests: p < 0.001 SCI versus normal and dogs with support; p < 0.01 SCI dogs versus lame dogs) but no differences between lame dogs and normal dogs, or between normal dogs with and without band support (Wilcoxon signed ranks test, p = 0.813).

The overall step cycle duration, as measured from left forelimb paw placements, was variable between individuals and within individuals at different speeds, as noted in the normal dogs. Examination of the normalised diagonal coupling in these dogs revealed that these were considerably – and significantly (Dunn's multiple comparison test: p < 0.001) – different from those of normal dogs (Fig. 4). In this group the overall diagonal coupling was a negative number, implying that the hind limb paw placement was delayed with respect to forelimb paw placement, therefore differing from the sequence found in normal dogs (see Fig. 4).

**Figure 5:**
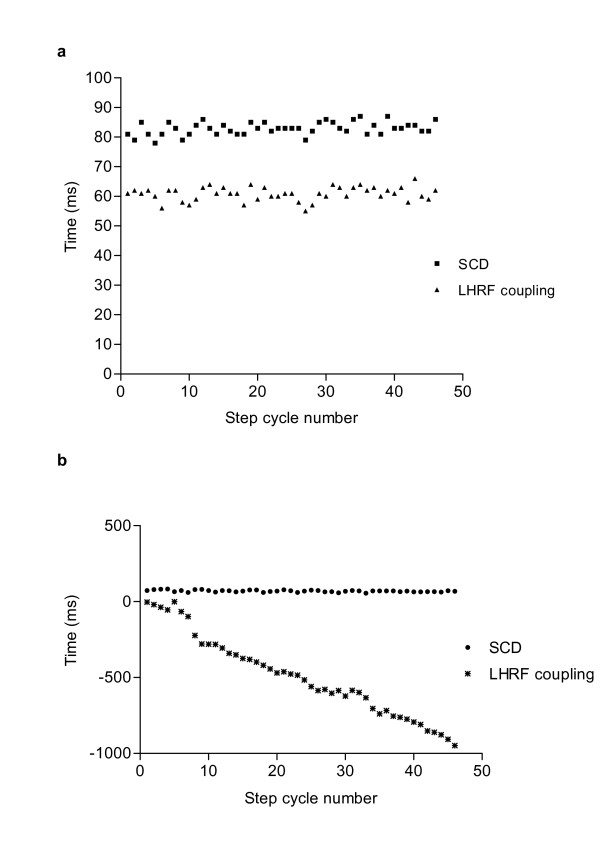
Representative plots of mean diagonal coupling interval in (**a**) a normal dog and (**b**) a SCI dog – note differences in y-axis scale. In the normal dog, although there is some variability in both step cycle duration (SCD) and diagonal coupling (LHRF) there is minimal change in this interval between step 1 and step 41. In contrast, in the SCI dog the interval is greatly prolonged, reaching ~800 ms during the same period of 40 step cycles.

We next examined the mean diagonal coupling intervals in this group (see example in Fig. 5), which was also markedly different from those of normal animals (Dunn's multiple comparison test: p < 0.001, and see Fig. 4). Again, although there was considerable inter-individual variation, the mean diagonal coupling interval was a negative figure, confirming that hindlimb paw placement was substantially delayed in relation to the forelimb paw placement. The profiles of the plotted diagonal coupling intervals at each step differed between individuals, with most dogs showing a gradual change in the coupling intervals (as shown in Fig. 5), but some exhibited a different pattern in which well-coordinated fore limb and hind limb paw placements were maintained for several consecutive steps but then one or more hindlimb steps were greatly delayed.

We next considered the possibility that the abdominal support band may have caused gait pattern alteration in SCI dogs. Therefore we compared the gait parameters of normal dogs as they walked with and without the abdominal support band. We found that in three of six examined dogs there was a significant effect on step cycle duration (paired T tests) and in one of six there was a significant effect on diagonal limb coupling (paired T tests). In contrast, comparison of the mean diagonal coupling intervals in normal dogs walking with and without abdominal band support revealed no significant effect (Wilcoxon signed rank test; p = 1.00) upon this parameter.

We concluded that although there was strong evidence that limb coupling intervals were significantly affected by SCI, this parameter could also be affected by abdominal band support and was highly variable amongst normal dogs (see section above). For this reason we considered that this parameter was not sufficiently robust to permit reliable comparisons between normal and SCI dogs. In contrast, the mean diagonal coupling interval was a highly conserved parameter with a very limited range in normal dogs, which was unaffected by provision of abdominal support and distinctly different between normal and SCI dogs.

### Dogs with orthopaedic disease

We next considered the possibility that orthopaedic causes of lameness might also similarly alter these measured descriptions of gait and present a confounding factor in future analysis of outcome after SCI. Therefore we next examined the gait of dogs that had orthopaedic disease in one limb; and a mean of 50 step cycles (range 20–80) was accepted for analysis in this group. This analysis confirmed that normalised diagonal coupling intervals were not significantly different between lame and normal dogs (Dunn's multiple comparison test; p > 0.05, see Fig. 4a). However, because of the caveats associated with use of simple limb coupling intervals (see above) we also examined the effects of orthopaedic disease on mean diagonal coupling intervals. Again, these were not significantly different between normal and lame dogs (Dunn's multiple comparison test; p > 0.05, see Fig. 4b).

## Discussion

Devising a method to quantify the effect of spinal cord injury on coordination between fore and hind limbs in dogs is a challenging task, since there are a great many variables that must be taken into account. Several previous studies investigating the effects of SCI in cats and rodents have concentrated on measures ranging from the recovered ability to take weight-bearing steps [29], to parameters relating to an individual limb, namely joint angles and electromyograph recordings [30,31], whilst others have focussed on forelimb-hindlimb coordination [27,32,33]. We show here that the mean diagonal coupling interval is unaffected by speed of walking, gait pattern, size of animal or orthopaedic disease. Therefore, it represents an extremely robust, yet simple, measure of coordination between fore and hind limbs. This will be of great value for future analysis of outcome of SCI treatments in dogs and other quadrupeds since we can now define in precise numerical terms the gait disturbance associated with thoracolumbar SCI thereby providing a replacement for methods of visual clinical scoring [25].

Previous studies that aimed to define fore-hind limb co-ordination in normal quadrupeds have also examined the correlation between forelimb and hindlimb step cycles [26,32-34]. However, their parameters were often derived by complicated and time consuming methods, and, as we show here, simple relationships between timing of fore and hind limb paw placement at each step are not sufficiently robust to summarise locomotion as 'normal' within a varied population of normal individual dogs. In our current study, by use of a treadmill to maintain a specified speed of locomotion and through computer-aided analysis, we have extended previous investigations to define mean diagonal pairing interval as a highly robust parameter with applicability beyond one species to include those within and outside a laboratory setting. Mean diagonal pairing interval also has the advantage of being a summary parameter, being the end result of analysis of a large number of step cycles and critically dependent on the rigidly time-locked sequence of forelimb and hindlimb step cycles. It would be expected that this parameter will be unaffected by alterations in gait during locomotion, although this remains to be tested.

Any method of gait analysis that relies on an animal walking on a treadmill could be criticised because of the possibility that apparent coordination is caused by the common substrate along which the fore and hind limbs are walking, causing the limbs of the two girdles to fall into an apparently entrained pattern simply due to the physical linkage. Similarly, the physical linkage between the fore and hind limb girdles or the spine could be a cause for apparent, but not 'real', coordination between fore and hind limbs. However, both these possible caveats are not compatible with the loss of coordination between fore and hind limbs that we have defined and quantified here in SCI dogs, and further supported by a previous report showing that mice with complete SCI can coordinate between the hindlimbs while walking on a treadmill, but not between the forelimbs and hindlimbs [27]. In addition, a previous study comparing overground with treadmill kinematics in the intact rat found only small differences in step cycle timing and joint angles, although coordination was not specifically examined, suggesting that treadmill walking is representative of normal locomotion [35]. Therefore we believe that coordinated walking on a treadmill in normal dogs represents a true observation and is not an epiphenomenon.

Provision of abdominal support is essential for evaluating the gait of animals with severe thoracolumbar SCI, since many are unable to stand or walk unaided, despite the ability to generate weak hindlimb movements. Therefore the question arises as to whether this weight support might confound interpretation of our data. Previous work in neonatal rats [36] and human patients [37,38] suggests that increasing the weight load on the pelvic limbs can increase their step frequency. However, these studies detected such effects when testing a range of weight support varying from zero to 75% of body weight, which is quite different from our study. Also, this previous work examined pelvic limb movement only, rather than its coordination with the thoracic limbs, for which there is no published information on the effects of partial weight support; there is the additional caveat that animals injured as neonates exhibit different recovery from those injured as adults [39]. In dogs of variable conformation of leg and spinal dimensions, such as the population we examine here, there is good reason to suppose that there will be some variability in the proportion of body weight 'normally' carried by the hindlimbs. In addition, the proportion of body weight carried by the hindlimbs will vary depending on the precise position on the treadmill and instant to instant acceleration or deceleration. Therefore we consider that our use of a support band to maintain a visually normal posture with adjustments to optimise stepping was the most reasonable solution, and, indeed, was shown not to affect forelimb-hindlimb coordination (measured by the mean diagonal coupling interval) in normal dogs.

In SCI dogs the mean diagonal coupling interval became a negative figure, implying that the hindlimbs were walking at a slower step cycle rate than the forelimbs. Such independent movement of the hindlimbs caudal to a thoracolumbar lesion is likely to be due to inherent rhythmogenicity of the lumbar spinal cord pattern generators, or to subsequent plasticity during recovery [40-44]. Our data would further suggest that although the hindlimbs are able to generate a stepping pattern independently of the forelimbs, this inherent pace is slower than that of the forelimbs. A similar result has been found in extensively lesioned cats [29,31,32]. The implication therefore is that, in normal animals, a more cranial instructive signal is required to accelerate the hindlimb movements, in contrast to the suggestions made from *in vitro *studies on neonatal rodent spinal cord that the hindlimbs are capable of regulating speed of activity in the forelimbs [45,46].

The neural substrate of coordination between fore and hindlimbs has been investigated in many previous studies and, theoretically at least, could be mediated by either propriospinal or supraspinal pathways. In rats and cats, it has been shown that destruction of the ventral and ventrolateral regions of the thoracolumbar spinal cord is sufficient to interrupt normal coordination between fore and hind limbs [32,47], correlating with *in vitro *data from neonatal rats that shows normal rhythmic activity in the isolated ventral third of the spinal cord [38]. Evidence also exists to suggest that the dorsolateral funiculus may be involved in fore-hindlimb coordination in the cat [31], and the corticospinal tract in monkeys [48]. In the SCI dogs examined here, the majority of lesions were ventral because of the nature of the inciting cause, as is also the case in most human SCI. Interestingly, it appears that long propriospinal axons may be less susceptible to contusive injury than descending tracts such as the corticospinal tract [49], thus providing a mechanism by which forelimb and hindlimb coordination could be preserved following even severe SCI in dogs.

Although forelimb-hindlimb coordination represents an important aspect of recovery after SCI in quadrupeds it is incomplete as a description of functional recovery, especially with regard to implications for therapy in humans since it is not a measure of goal-directed (implying supraspinally-driven) behaviour. For that reason, it may appear that measures of coordination between fore and hind limbs have limited impact as a means to define the efficacy of an intervention in human SCI patients. However, there is considerable evidence that the human nervous system retains much of the coordination between 'fore' and 'hind' limb coordination observed in quadrupeds [50,51], suggesting the possibility that physical tasks in which human subjects were required to coordinate effort between their unaffected arms and their paretic legs might be devised. Nevertheless, the major importance of devising a measure of fore-hind coordination is to provide a robustly quantifiable parameter for determining the effect of an intervention in pre-human trials. Therefore, the ability to analyse the gait of dogs in great detail using this technique will have important implications for determining the rate of progress towards human clinical trials.

## Methods

### Equipment set-up

**Figure 6:**
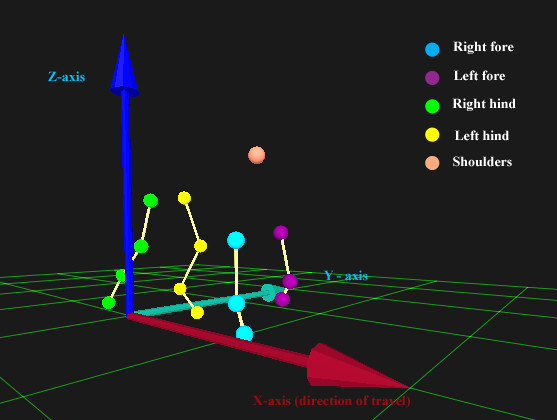
'Screenshot' image obtained from Qualisys software incorporating a representation of a dog walking on the treadmill and the orientation of the x-, y- and z- planes in relation to the direction of forward motion of the dog.

Four infrared motion capture cameras with a recording frequency of 100 Hz (Qualisys, Sweden) were positioned around a standard ex-gymnasium treadmill and calibrated to permit recording from the entire belt surface. We defined the sagittal plane (forward and backwards) as the x-plane, the vertical plane as the y-plane and the z-plane as lateral movements (Fig. 6). Dogs were placed on the treadmill belt, and led by a handler positioned at the front of the treadmill. 10 mm reflective markers were attached using double-sided adhesive tape to the skin, clipped as necessary, overlying specific anatomical landmarks: lateral fifth phalange, lateral humeral epicondyle, ulnar styloid process, greater trochanter of the femur, lateral femoral epicondyle, lateral malleolus of the tibia, and the interscapular region dorsal to vertebra C7.

Spinal cord injured dogs required additional lateral support because they were unable to walk without swaying and falling to the side. Therefore lateral stability was assured by passing a padded band under the caudal abdomen and attaching it to the frame of the treadmill. Tension in the support band was adjusted so that the thoracolumbar part of the vertebral column was parallel to the treadmill belt surface and minor adjustments were made to maximise the intensity of stepping movements. Since ~60% of body weight is supported through the forelimbs of normal dogs and there is variation from instant to instant in the precise loading of the limbs during acceleration and deceleration [52] we estimate the load bearing of the band to be between ~10 and 35% of body weight during a step cycle, depending on the degree of lateral sway.

### Recording

Dogs were initially accustomed to walking on the treadmill whilst held and encouraged by the handler who was positioned at the front of the treadmill. Introductory speeds of 0.3 – 1 kmh^-1 ^were used to allow each individual dog to become familiarised with walking on the moving belt and to the noise of the motor. The speed was then increased gradually until the dog was walking with a consistent gait and with minimal variation in pace or lateral movement; this varied from 1 to 3 kmh^-1^, depending upon the dog's size. Dogs were then allowed to continue at this speed for ten minutes after which the reflective markers were attached as described above. During recording, the treadmill speed was set to the speed determined previously, at which the dog was walking consistently, and 60 seconds of motion was recorded. The treadmill speed was then increased and recordings repeated at a variety of speeds. In some normal animals we examined the effect of applying belt support, to act as a control for this means of assistance that was essential for dogs that had spinal cord injuries.

### Data analysis

Processing of the recorded images was initially carried out using Qualisys Track Manager software (Qualisys QTM, Sweden). The 15 individual markers were identified and labelled to construct a 3D stick-diagram representation of the dog. Visual examination of lateral and forward movement was displayed in QTM graphical plots of y- (lateral) and x- (sagittal) plane position and used to exclude sections of data in which a dog was not walking consistently (recognised by abrupt irregularity in the plot, indicating acceleration or deceleration, or sudden movement to one side). Subsequent analysis of coordination was focussed on data obtained from the paws only (phalangeal markers). Positional data from x-plane plots of each paw were exported from QTM into Matlab (Release 14, student version) and timing and value of the maximal and minimal x-plane points of each step cycle were extracted using a custom-written script. This produced single-column matrices of data describing the temporal and positional occurrence of the extremes of sagittal paw movement. Although maxima and minima represent approximations to the start and end of the stance phase they were used here to permit standardised comparison between normal and SCI dogs, since SCI dogs were not consistently able to lift the paws from the treadmill and therefore did not have distinct 'swing' and 'stance' phases. The resulting data was then manipulated to calculate step cycle features using standard Matlab matrix addition and subtraction functions:

#### Step cycle duration (see Fig. 1)

Overall step cycle duration was calculated using the left front paw as a benchmark, by measuring the time elapsed between one maximal x-plane position point and the next.

#### Limb coupling (see Fig. 1)

The interval between individual movements of specific pairs of limbs was derived by calculating the time between maximal x-plane positions of the relevant limbs. Thus, *homolateral coupling *– the time between initiation of stance phase in each of the fore and hind limbs on one side of the animal – was derived by calculating the time difference between maximal x-position of a hindlimb from that of the respective forelimb at each step; *diagonal coupling *was similarly calculated, but using diagonal pairs of forelimbs and hindlimbs. Both these parameters could be calculated to be positive or negative numbers, reflecting the sequence of fore and hind paw placement in each step. This data was then plotted as line graphs against step number to demonstrate inter-cycle variations in the timing features of each individual step cycle.

To allow comparison between different individuals and between groups of dogs that had incurred different injuries we also expressed limb coupling time as a proportion of total step cycle duration – the 'normalised' limb coupling time. This parameter was thus independent of step-cycle duration, which was variable depending upon the treadmill speed and individual conformation.

#### Mean diagonal coupling interval (see Fig. 3)

As an extension of the limb coupling parameters described above, we used Matlab to calculate a single numerical summary measure of the average length of time per step that the right hind limb paw placement was delayed after the left forelimb paw placement during the entire period of recording. [Although we arbitrarily studied the left fore-right diagonal coupling, any of the inter-girdle limb pairs could have been used.]

To derive this measure, counts of step number were made by the Matlab program's detection of the maximal and minimal x-plane positions during each step cycle, meaning that a record was kept of the number of steps made by each fore and hind paw. The accumulated time difference between the '***n*th**' fore and '***n*th**' hind limb paw placement was then subtracted from that between the corresponding **first **fore and **first **hind limb paw placement to provide the total delay across the whole recording period. This number was then divided by the number of steps. We use the term '*mean diagonal coupling interval*' to describe this measurement, and which is defined by the equation:

Mean diagonal coupling Interval = Tn – T1/number of steps

Where Tn is the interval between the '*n*th' right hind limb paw and '*n*th' left fore limb paw placement; T1 is the interval between the first right hind limb paw and first left fore limb paw placement. The derivation of this parameter is explained graphically in Figure 3 for a normal and a SCI dog.

In view of this calculation method, normal dogs were predicted to have a numerical value very close to zero, corresponding to rigidly time-locked coordination of fore and hind limb movement in whatever gait pattern they were using.

### Dog selection

Three groups of dogs were examined:

#### Group 1

Normal dogs (n = 11) that were free of orthopaedic and neurological disease and were volunteered by their owners for use in this study (owned by members of the Department). These dogs were of both sexes, and were representative of the wide variety of breeds and conformation in the dog population; tibial length (measured from lateral malleolus to lateral tibial condyle – used as an easily-measured surrogate of full limb length) varied from 6.5 to 26 cm (mean 15.9 cm).

#### Group 2

These were dogs (n = 12) diagnosed by MRI or other imaging techniques to have a defined region of traumatic thoracolumbar spinal cord injury and were presented for treatment in the Departmental Veterinary Hospital; their clinical details are recorded in Table 1. This group included dogs that had sensory 'incomplete' or 'complete' injuries, closely corresponding to ASIA grades A and C in human patients (in veterinary medicine sensory complete animals are assumed to have no volitional control over the hindlimbs). Injuries were located between T11 and L2 spinal cord segments and resulted from mixed contusive/compressive lesions: two dogs had suffered vertebral fractures, nine had suffered acute intervertebral disc extrusion (a stereotypical mixed compressive/contusive lesion common in dogs) and one was examined after surgery for removal of an intramedullary spinal cord tumour (a nephroblastoma). During the period of recording for this study none of these animals could walk without assistance and were therefore supported on the treadmill as described above. The tibial lengths in these dogs ranged from 7–22 cm (mean 13.3 cm).

#### Group 3

Dogs that were presented for treatment for orthopaedic conditions in the Departmental veterinary Hospital, but free from neurological disease (n = 8). These dogs were assessed to determine whether disruption in gait patterns caused by non-neurological conditions would affect each of the measures of forelimb-hindlimb coordination examined in this study. The cause of lameness in these dogs was diagnosed to be ruptured anterior cruciate ligament in four cases, elbow osteoarthritis in two cases, plus one each of patellar luxation and femoral head and neck excision. Tibial lengths in this group ranged between 9–23 cm (mean 16.8). The clinical details of these animals are summarised in Table 2.

**Table 2:** Clinical details of dogs with orthopaedic injuries

Dog ID	Tibial length (cm)	Lesion type
1	17	Left luxating patella
2	10	Left CCLR
3	23	Left femoral head & neck
4	18	Left CCLR
5	9	Right CCLR
6	21	Right elbow osteoarthritis
7	22	Left CCLR
8	14	Right elbow osteoarthritis

CCLR, cranial cruciate ligament rupture.

### Statistical analysis

Data acquired in QTM were transferred as numerical data into Matlab. A custom-written script was used to extract the data points of interest and standard matrix addition or subtraction was used to calculate time intervals and position as described above. The resulting data was assembled in Excel spreadsheets and transferred into GraphPad Prism (Version 3.0 for Windows) for statistical analysis.

For each animal there were columns of data listing the step cycle duration, homolateral and diagonal coupling intervals *for each step*. ANOVA was used to compare the range and variability of each of these parameters amongst the different individual normal dogs. *Post hoc *tests, using Tukey's test, were applied where appropriate. Only a single cumulative diagonal coupling interval was available from each step cycle series, meaning that comparison was made only between groups of animals. All groups (normal, SCI and orthopaedic) were compared together using the Kruskal-Wallis test, and *post hoc *Dunn's tests where appropriate used to determine specific differences where significance was detected. Paired Student's T test and Wilcoxon signed ranks test were used to compare data derived from normal animals walking with and without abdominal band support.

In each instance in which multiple groups were eventually compared we used ANOVA or the Kruskal-Wallis test to compare all the groups initially, although the results are reported sequentially. Where this occurs we have reported results of *post hoc *tests, full details are given in figure legends. Significance was assumed when p < 0.05.

## Competing interests

The author(s) declares that there are no competing interests.

## Authors' contributions

LH carried out the bulk of the gait recordings, produced the Matlab analysis and prepared illustrations for publication. RJMF participated in study design, direction and supervision. NDJ conceived the study, participated in design, carried out the statistical analysis and prepared the manuscript. All authors read and approved the final manuscript.
